# Decoding CBME through a longitudinal faculty development program: A comparative study

**DOI:** 10.6026/973206300212206

**Published:** 2025-07-31

**Authors:** Sudhanshu Agrawal, Rupa Singh, Abhinav Singh, Ashish Goel

**Affiliations:** 1 Department of Physiology, Mahatma Vidur Autonomous State Medical College, Bijnor, Uttar Pradesh, India; 2 Department of Physiology, Shri Guru Ram Rai Institute of Medical & Health Sciences, Dehradun, Uttarakhand, India

**Keywords:** Faculty Development Programs (FDP), CISP, competency-based medical education (CBME), pre- test, post- test, feedback, longitudinal

## Abstract

National Medical Commission (NMC) conducts two-day CISP workshop under Faculty Development Programs (FDP) for CBME but short duration may not be enough for proper faculty improvement. In our study, 62 faculty (CISP trained and non-trained) underwent longitudinal FDP with CBME modules for three months with weekly sessions, pre-post tests and feedback were taken. Initially trained faculty performed better but with repeated sessions, non-trained also improved and both groups showed similar post-test results and feedback trend. So, CISP should be done as longitudinal program to save time and manpower and to improve CBME understanding in faculty.

## Background:

The National Medical Commission (NMC) has brought many reforms in medical education in India. ROME was started in 1977, followed by Graduate Medical Education Regulations in 1997, and Vision 2015 which promoted CBME approach [1, 2]. To support this, NMC started various Faculty Development Programs (FDP) like Basic Course, CISP and Advance Course to train faculty for CBME [3, 4- 5]. These FDPs focus on teaching methods, assessment, communication and professionalism [2, 3, 4- 5]. CISP is usually conducted as two days' workshop to orient faculty towards CBME principles [6, 7]. But short workshops may not be enough to bring long-term behavior or attitude change in faculty. Longitudinal FDP can help by giving repeated sessions, reflection, practice and feedback to improve teaching skills [8, 9- 10]. Studies by Knight *et al.* and Schlair *et al.* also showed that longitudinal FDP improved learner-centered teaching, feedback skills and confidence among faculty [11, 12]. Therefore, it is of interest to compare the effectiveness of longitudinal FDP based on CBME modules between CISP-trained and non-trained faculty. We planned this program to assess how much both groups understood CBME concepts and whether they could improve their learning and skills after completing the sessions.

## Materials and Methods:

This longitudinal study was carried out from November 2020 to August 2021 in National Capital Region Institute of Medical Sciences (previously MSY Medical College), Meerut and India. Prior to study, approval was taken from Institutional Research Committee and Ethical Committee of NCRIMS. Total 65 faculty members were selected by purposive non-randomized sampling after giving information sheet and taking proper informed consent. Out of them, 31 faculties were already CISP trained and 34 were non-trained. All participants undergone three months longitudinal faculty development program (March to May 2021) which included six modules of CBME based CISP training. Pre and post-test questioners were given for both qualitative (open-ended) and quantitative (MCQ based) assessment. Feedback forms were also provided after every session. These tools were prepared by researchers after going through literature [8, 9-10] and validated by Medical Education Unit and Ethics Committee experts. Related literature was also given to all participants in soft copy format. Data was analyzed qualitatively and quantitatively. For statistical analysis GraphPad InStat 3.1 software was used.

## Results:

Out of total 65 faculty members enrolled, 62 participants completed all six sessions, which includes 30 CISP-trained and 32 non-trained faculties. It was seen that initially the CISP-trained faculty given more relevant responses in pre-test for CBME, deriving objectives and elective modules when compared to non-trained faculty. But as the sessions progressed, both groups gave similar responses in post-test. For AETCOM, alignment and integration and assessment modules, there was only little difference in pre-test responses and in post-test both groups were almost similar. The pre-test scores were significantly better for CISP-trained group for CBME (25 ± 0.45 vs. 15.8 ± 0.65; p < 0.001), deriving objectives (26.2 ± 0.48 vs. 19.2 ± 1.05; p < 0.001) and electives (24.8 ± 0.31 vs. 21.3 ± 0.49; p < 0.001) (Table 1 - see PDF). For other modules like AETCOM, alignment and integration and assessment there was no much significant difference. In post-test, both groups performed almost similar, CBME module had scores 29 ± 0.37 for trained group and 29.8 ± 0.31 for non-trained (p = ns). In electives module, slight improvement was seen in non-trained faculty (31.5 ± 0.34 vs. 30 ± 0.00; p < 0.05). It was noted that with continuation of study, non-trained participants shown progressive improvement in self-perceived knowledge and relevance. By end of sessions, their feedback scores were better than trained group for CBME module (31.8 ± 0.25 vs. 29.8 ± 0.25; p < 0.001) (Table 2 - see PDF). Same pattern was seen in other modules also. Majority of participants from both groups expressed that FDP should be continued as longitudinal program instead of two days' workshop.

## Discussion:

The present study compared the effectiveness of longitudinal FDP based on CBME modules between CISP-trained and non-trained faculty. It was seen that though trained faculty performed better in pre-test for CBME (25 ± 0.45 vs. 15.8 ± 0.65; p < 0.001), deriving objectives (26.2 ± 0.48 vs. 19.2 ± 1.05; p < 0.001) and electives (24.8 ± 0.31 vs. 21.3 ± 0.49; p < 0.001), this difference reduced by post-test, where scores became almost similar like CBME (29 ± 0.37 vs. 29.8 ± 0.31; p = ns). This shows that regular and repeated exposure in longitudinal FDP can help non-trained faculty to reach same level. Similar pattern seen by Knight *et al.* where longitudinal FDP improved 14 out of 15 teaching behaviors significantly (p < 0.05) [11]. Schlair *et al.* also reported sustained improvement in feedback and observation skills with longitudinal exposure [12]. Our feedback also showed same pattern where non-trained faculty gained more self-confidence and relevance with time. Their self-perceived knowledge scores were higher than trained group for CBME (31.8 ± 0.25 vs. 29.8 ± 0.25; p < 0.001), electives (31.8 ± 0.25 vs. 30.0 ± 0.00; p < 0.01) and AETCOM (32.0 ± 0.00 vs. 30.0 ± 0.00; p < 0.05). This reflects better professional self-efficacy which was seen in previous FDP studies also. Our findings also match with global data. Bilal *et al.* reported pooled effect size of 0.73 (z = 4.46; p < 0.05) showing FDP improves knowledge and skills significantly [13]. Owolabi *et al.* showed MCQ item quality improved after longitudinal FDP with Cronbach's alpha rising from 0.51 to 0.84 (p < 0.0001) [14]. Ayub *et al.* also found MCQ writing improved significantly with repeated FDP sessions (χ^2^ = 955.86; p < 0.05) [15]. Globally Harvard fellowship by Newman *et al.* showed that longitudinal FDP fellows performed better than control group in 6 out of 10 academic career areas like promotions, leadership and committee work and funding [16].

Same pattern we also observed where objective scores and confidence improved after longitudinal training. Apart from numbers, many theories also explain why longitudinal FDP works better than short workshops [17]. Knowles adult learning theory says adults learn better with self-direction, reflection, active participation and real-life application [18]. Our FDP used similar cyclical structure with repeated sessions, peer discussions and feedback to improve learning and confidence. Ahmed *et al.* suggested 5x2-D model, which shows FDP should be continuous process with reflection and improvement cycles, not one-time [19]. Our design also followed this approach. Global review by Kohan *et al.* summarised 119 studies and concluded FDPs should cover full faculty roles including mentorship, leadership and assessment, not just content teaching [20]. Our multi-module FDP included these aspects, leading to overall faculty development. These global findings are very relevant to Indian CBME scenario. As Gopalakrishnan *et al.* and Soundariya *et al.* mentioned, CBME in India is facing challenges like limited resources, lack of faculty readiness and gaps in assessment skills [21, 22]. Our study shows longitudinal FDP can help by building skills slowly, reducing variation between trained and untrained faculty and creating CBME-ready teaching teams. Rahman *et al.* also showed FDP participants performed better than non-participants in teaching, curriculum work and research and technology use [17]. This highlights FDP benefits beyond just classroom teaching. Our results prove that while CISP training helps initially, longitudinal FDP is needed to bring all faculties to same level of competence and confidence. This is not only knowledge gain but also improvement in attitude, behavior and professional identity, which CBME system demands. Still our study has few limitations. Pre and post-test survey is useful but can have internal validity issues and depends on participants' memory [8, 12]. Sample size is also small, so in future, we will like to extend this study with more faculties from different institutes.

## Conclusion:

The study shows that longitudinal FDP is useful for improving CBME understanding in both CISP trained and non-trained faculty. Though trained group had advantage in beginning, non-trained also improved with regular sessions. Majority of participants preferred longitudinal model over two day's workshop. So, CISP can be planned as longitudinal program to improve faculty competence in CBME.
